# Stem Cells in the Periodontium—Anatomically Related Yet Physiologically Diverse

**DOI:** 10.1055/s-0042-1759487

**Published:** 2022-12-31

**Authors:** Deepa Ponnaiyan, Roshan R. Rughwani, Dhayanand John Victor, Ganesh Shetty

**Affiliations:** 1 Department of Periodontics and Oral Implantology, SRM Dental College and Hospital, Ramapuram, Chennai, Tamil Nadu, India; 2 Dental and Orthodontic Clinic, Bangalore, Karnataka, India

**Keywords:** 3D-bioprinting, exosomes, immune phenotype, periodontal regeneration, periodontal stem cells, regenerative medicine, stem cells, tissue engineering

## Abstract

Periodontitis is a complex chronic disease discernible by the deterioration of periodontal tissue. The goal of periodontal therapy is to achieve complete tissue regeneration, and one of the most promising treatment options is to harness the regenerative potential of stem cells available within the periodontal complex. Periodontal ligament stem cells, gingival mesenchymal stem cells, oral periosteal stem cells, and dental follicle stem cells have structural similarities, but their immunological responses and features differ. The qualities of diverse periodontal stem cells, their immune-modulatory effects, and variances in their phenotypes and characteristics will be discussed in this review. Although there is evidence on each stem cell population in the periodontium, understanding the differences in markers expressed, the various research conducted so far on their regenerative potential, will help in understanding which stem cell population will be a better candidate for tissue engineering. The possibility of selecting the most amenable stem cell population for optimal periodontal regeneration and the development and current application of superior tissue engineering treatment options such as autologous transplantation, three-dimensional bioengineered scaffolds, dental stem cell-derived extracellular vesicles will be explored.

## Introduction

Periodontal disease is a chronic inflammatory disease characterized by a dysregulation of balance between the native oral commensals and the pathogenic microorganisms, leading to activation of the inflammatory cascade thereby causing host mediated destruction of the periodontal soft and hard tissues. 1 Nonsurgical periodontal therapy (NSPT) primarily aims at regulating the immune-inflammatory profile by mechanical debridement. Despite this, in approximately 67% of instances, disease persists even after NSPT owing to areas of persistent pockets that do not allow complete resolution of inflammation, thereby warranting surgical treatment. 2

Traditional surgical periodontal therapies rely on synthetic materials and biological agents for regeneration, although their effectiveness is debatable due to lack of histological evidence of regeneration. 3 Tissue engineering with a triad of cells cultivated on a scaffold with suitable biophysical and chemical cues to finally rebuild the lost tissues has been proposed for attaining optimal regeneration. 4

Stem cells have been considered a promising approach for regeneration as they have unique properties of stemness, migration, differentiation, and immune modulation. 5 Traditionally, stem cells have been harvested from the dental pulp and the exfoliated deciduous teeth; however, recently the use of stem cells sourced from the periodontium has been advocated as they are a reservoir of highly undifferentiated cells that can migrate to regenerate the lost periodontium. Mesenchymal stem cells from periodontal tissues such as periodontal ligament stem cells (PDLSCs), gingival mesenchymal stem cells (GMSCs), oral periosteal stem cells (OPSCs), and dental follicle stem cells (DFSCs) have been tested with varying results *in vitro* and *in vivo* for regeneration. The best possible stem cell needs to be assessed and compared to aid in the selection of the candidate cell to achieve complete regeneration. This review summarizes the properties of each unique stem cell population harvested within the periodontium and their regenerative potential.

## Origin and Distinct Phenotypes of Stem Cells within the Periodontium

**Fig. 1:**
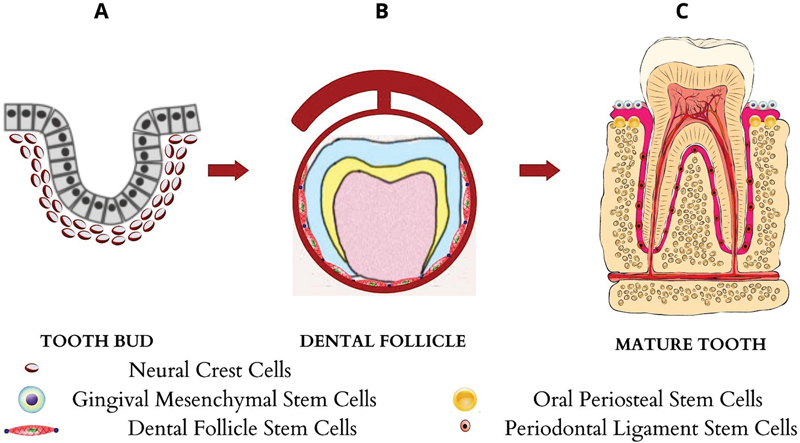
Origin and development of stem cells within the periodontium. ( **A** ) The neural crest ectomesenchyme and the neural crest cells—The thickening of ectomesenchyme with migration of neural epithelial cells form a tooth bud. ( **B** ) Condensation of mesenchyme to form dental papilla and follicle giving rise to dental follicle stem cells. Inner layer of follicle (dental follicle proper) gives rise to periodontal ligament cells, the denotgingival fiber system harboring periodontal ligament stem cells (PDLSCs) and alveolar bone harboring oral periosteal stem cells (OPSCs), outer layer of follicle (perifollicular mesenchyme) forms gingival lamina propria that harbors gingival mesenchymal stem cells (GMSCs). ( **C** ) Mature tooth showing location of PDLSCs, GMSCs, and OPSCs.

The periodontal complex's postnatal root development that parallels the tooth growth process heightens the possibility of a bountiful supply of dental stem cells that are more embryonic in nature than other dental stem cell sources. The nomenclature of periodontal MSCs is strongly linked to their tissue origins ( Fig. 1 ). PDLSCs are produced from ectomesenchymal cells originating from the neural crest and are principally extracted from the mid-third of the root surface post extraction of permanent teeth. 6 The stem cells isolated from root surface are termed root surface derived PDLSCs (r-PDLSCs) and the stem cells isolated from the tissue collected from the bone surface are called alveolar socket derived PDLSCs (a-PDLSCs). It has been observed that a-PDLSCs retain more proliferative capacity, high osteogenic, and adipogenic potential compared to r-PDLSCs. 7 Seo et al, 2004, found that PDLSCs have the ability to differentiate into periodontal ligament, alveolar bone, cementum, peripheral nerves, and even blood vessels. PDLSCs are found in the periodontal ligament and the developing follicle of permanent teeth. 8

Gingiva, the most important periodontal organ, is the next source of stem cells that originate from the neural crest and even from the bone marrow. GMSCs are extracted from gingival tissue samples acquired during gingivectomy procedures and de-epithelialized to leave only connective tissue. 9 Neural-crest-derived GMSCs (N-GMSCs) and mesoderm-derived GMSCs (M-GMSCs) are two subpopulations of gingival mesenchymal stem/progenitor cells, with N-GMSCs having a stronger ability to develop into neural cells than M-GMSCs. 10

Alternatively, a loose ectomesenchyme-derived connective tissue called dental follicle surrounds the enamel organ and dental papilla of the growing tooth germ. The DFSCs are comprised of a series of pluripotent stem cells formed from neural crest cells originating from the ectoderm. 11 Morsczeck was the first to discover that periodontal tissue progenitors were present in dental follicular cells, known to regulate osteoclastogenesis and osteogenesis that is vital for tooth eruption coordination, in addition to its role in periodontal development. 12 OPSCs are derived from the periosteum, a complex structure that includes undifferentiated mesenchymal cells and envelopes the bone. 13 The periosteal stem cells regulate chondrogenesis and osteogenesis that can be exploited for the maintenance of bone mass in both physiological remodeling and in periodontal surgical healing process.

Dental tissues in the craniofacial complex have become a readily available source of MSCs with multilineage differentiation capabilities comparable to bone marrow MSCs. 14 Despite the fact that there are numerous dental stem cell populations, periodontal stem cells have recently attracted a lot of attention because they are native to the periodontal complex and can be induced to accomplish regeneration. 15 MSCs from the periodontal complex have morphology and marker expression that are very similar to fibroblasts, despite the fact that they only make up around 1% of the cell culture population. There are two main methods for isolating SCs from the periodontium, and both have been successfully employed to isolate MSCs from oral tissues, including gingiva for cell culture.

The first method entails growing cells from a tissue sample in plastic-adherent fibroblast: explant culture method or by enzymatic release method. 16 The second method includes MSCs sorting from parental fibroblast cultures or cell population enzymatically liberated directly from connective tissue biopsy based on a panel of preselected cell surface markers utilized to isolate MSCs from oral and other tissues. This method of prospective separation is based on the ability of the chosen markers to accurately identify MSCs. 17 Based on these above techniques, MSCs can be identified within the periodontium and probably the biggest colonies may be identified as putative MSCs with highest potential for proliferation and self-renewal.

### Distinct Phenotypes of Stem Cells within the Periodontium:

The periodontal stem cells originate from closely related tissues; however, there is wide difference in the type of markers they express, which determines the unique phenotype and differentiation capacity of each of these stem cells ( Table 1 ).

**Table 1:** Phenotypic expression and regenerative potential of periodontal stem cells

Stem cell type	Markers expressed			*In vivo* tissue formation	*In vitro* tissue formation	Reference
	Neural crest	Embryonic	CD antigen (+) / (-)			
PDLSC	Sox 10, P75NTvnR, Snail, Twist, Sox-9, CD49d	Nanog, Sox 2, SSEA4 Oct 4, Klf4	CD-9 + , CD-10 + , CD-13 + , CD-29 + , CD-44 + , CD-59 + , CD-73 + , CD-90 + , CD-105 + , CD-106 + , CD-146 + , CD-166+ CD14-, CD31-, CD34-,CD45-	Cementum, PDL, adipose, dentin, bone	Osteo, adipo, chondro, myo, neuro, cardiomyo, HLC, melanocyte	18
GMSC	Snail1, Twist 1, Sox 9, NES, FoxD3, PAX3	Nanog, Sox-2 SSEA3	CD-29 + , CD-44 + , CD-73 + , CD-90 + , CD-105 + , CD-106 + , CD-146 + , CD-166+ CD34-, CD45-,CD117-	Cartilage, bone, muscle	Adiopo, chondro, osteo, neuro, endothelial cells	. 19
DFSC	HNK1, NES, P75NTR, Nestin, βIII-Tubulin	Oct 4, Sox 2, Nanog	CD-13 + ,CD-29 + , CD-9 + , CD10 + , CD-44 + , CD-59 + , D-53 + ,CD-73 + ,CD-90 + ,CD-105 + , CD106 + ,CD146 + , CD166 + ,CD34-, CD45-,CD31-, CD117-, CD14-	PDL like, cementum like, alveolar bone	Osteo, adipo, chondro, myo, neuro, cemento, odonto, HLCs	. 12
OPSC	Nestin, NG2	Hox-11, Nanog	CD-13, CD-29, CD-44, CD-71, CD-73, CD-146, CD34-, CD45-, CD105-,CD166-, D117-, CD90-	Bone	Osteogenic, neurogenic chondrogenic	20

Abbreviations: adipo, adipocyte; cardiomyo, cardiomyocyte; CD, cluster of differentiation; cemento, cementoblast; chondro, chondrocyte; FOXD3, forkhead box D3; DFSC, dental follicle stem cell; GMSC, gingival mesenchymal stem cell; Klf4, Kruppel-like factor 4; HLC, hepatocyte like cells, myo, myoblast; HNK-1, human natural killer 1; Hox, homeobox; Nanog, nanog homeobox; NES, neuro epithelial stem cell protein; neuro, neuronal cell; NG2, neuron glial antigen 2; Nestin, neuroepithelial stem cell protein; odonto, odontoblast; OPSC, oral periosteal stem cell; osteo, osteoblast; Oct 4, octamer 4, PAX3, paired box 3; PDLSC, periodontal ligament stem cell; PDL, periodontal ligament; P75NTR, p75-neurotrophin receptor; Snail-1, Slug- Zinc finger protein; Sox-SRY-related HMG-box genes; SSEA4, stage specific embryonic antigen; Twist 1, twist related protein 1.

+ - positive expression, - negative expression; elevated expression, decreased expression.

The PDLSCs, for instance, are found to be more of adult mesenchymal-like as they express the entire range of MSC markers and less of neural crest markers apart from sharing similar phenotype to pericytes as seen by positive expression of CD146, neural/glial antigen-2, and CD140B. 21

The majority of putative stem cell markers have been found to be expressed by GMSCs, and it has been observed that GMSCs express high levels of the embryonic stem cell markers Oct 4, Nanog, and SSEA3, which are essential for maintaining progenitor status, when cultured in three-dimensional (3D) scaffolds primed with ascorbic acid. 22 The significant expression of pluripotent markers in GMSCs indicates that gingival stem cells have the propensity for regeneration and incorporate stem cells that constitute pluripotent properties. It is discovered that the DFSCs are more embryonic in origin and express Oct 4, but only infrequently Nanog and CD90 that demonstrate the increased heterogeneity and neural crest genesis of DFSCs. 23

The periosteum offers mechanical support while also acting as a major source of progenitor cells and growth factors for bone regeneration. 24 Further, OPSCs showed high expression of CD-73, CD-90, CD-105, and CD-29, whereas hematological markers CD-45 and CD-34 were not expressed. 25 OPSCs have a low level of CD-117 surface marker positivity, which is a stem antigen expressed on MSCs that are not yet committed to bone phenotype. 26 OPSCs are distinguished from other types of stem cells by the selective expression of cathepsin K in the periosteum as early as embryonic day 14. 27

Overall, the stem cells of periodontal origin exhibit a wide spectrum of characteristics that can be harnessed for optimal regeneration based on a case-to-case scenario. While the GMSCs exhibit a wide range of embryonic markers, selecting a PDLSC or an OPSC for extensive periodontal regeneration would be more preferred as they have unique properties such as expression of greater osteogenic and chondrogenic differentiation markers that can achieve cementum and periodontal ligament like structures when transplanted in animal models, thereby making them a superior choice when compared to the application of GMSCs. 28 Further, the data for PDLSCs and OPSCs to form bone suggest that they can be adequately osteogenic only when they are used in a combination with suitable grafting materials until which their regenerative potentials cannot be tapped appropriately. On the other hand, the DFSCs have been shown to provide a suitable microenvironment for enhanced regeneration of PDLSCs *in vivo* , thereby highlighting the adjunctive role of DFSCs by acting as a scaffold in facilitating the differentiation of other types of stem cells. 29

## Inflammatory Environment and Periodontal Stem Cells—The Bidirectional Link

**Fig. 2:**
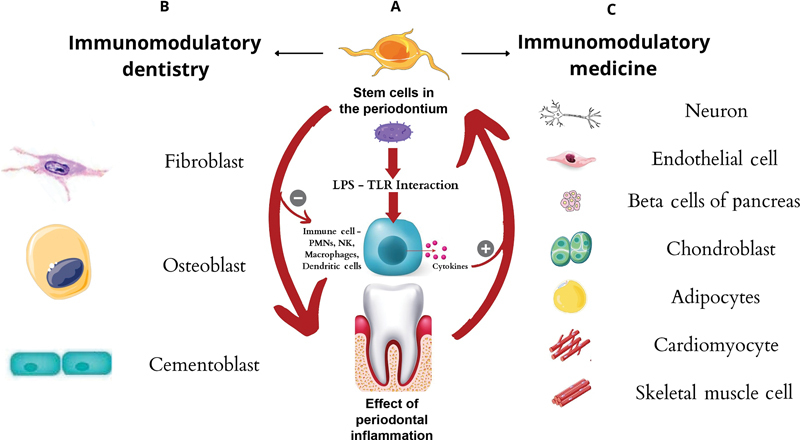
Immunomodulation and regeneration potential of periodontal stem cells. ( **A** ) Inflammation and stem cells “The Bidirectional Effect” affecting their differentiation. ( **B** ) Immunomodulatory dentistry—Regeneration of periodontal tissues like bone, cementum, and periodontal ligament. ( **C** ) Immunomodulatory medicine—regeneration into various tissues of the body.

The behavior of stems cells in an inflammatory environment differs markedly from the healthy state as inflammation affects the stem cells and in turn the stem cells exert immune-modulatory properties in an inflammatory microenvironment. 19 30 Understanding this bidirectional link of how periodontal inflammation can influence stem cells and how they interact in an exacerbated immune inflammatory environment can help devise future regenerative strategies ( Fig. 2 ).

### Effect of Periodontal inflammation on Stem Cells

In periodontal disease, gram-negative bacteria predominantly Porphyromonas gingivalis, Treponema denticola, and Tannerella forsythia are key microbial regulators of the disease, causing increased expression of inflammatory mediators and adhesion molecules triggering an inflammatory cascade there by altering the immune phenotype of the stem cells in the periodontally destructed sites. This inflammatory response acts as a regulator of tissue stemness either by directly affecting periodontal tissue stem cells or by shifting differentiated cells toward a stem cell like characteristic. The balance in this inflammatory response and mediated stemness is a critical driver of either maintaining tissue integrity or promoting aberrant homeostasis and disease. PDSLCs have been studied to have scope to regenerate the supporting structures as long-term stimulation of PDLSCs by *P. gingivalis* lipopolysaccharide (LPS) resulted in increase in cellular cytokine release compared to GMSCs. It is also suggested that LPS restrains the osteoblast differentiation by impairment of alkaline phosphatase activity and mineral deposition in DFSCs and PDLSCs. 31 Activation of TLR2 also caused increased proliferation of OPSCs. 32 On the contrary, studies also reported that *P. gingivalis* LPS treatment did not alter the cell viability of both OPSCs and DFSCs. This data suggest that the activation of inflammatory cascade has a role in the stimulation of various periodontal stem cells; however, the availability of contradictory evidences questions the phenomenon of this effect thereby warranting the need of future research.

### Immunomodulatory Effect of Periodontal Stem Cells on Inflammatory Environment

The effect of periodontal inflammation could sensitize the stem cells; however, recent data suggest that these stem cells also exert an immunomodulatory effect that explains the opposing link between SCs of periodontal origin and local inflammation. Immunomodulation is characterized by an induction, amplification, attenuation, or prevention of the functioning of the immune system by the activity of an immunomodulator such as the periodontal stem cells.

PDLSCs induce the secretion of tumor growth factor-β, indoleamine-2,3 di-oxygenase-1 and hepatocyte growth factor that have immunomodulatory effect on periodontal regeneration. 33 PDLSCs also alter the innate immune response by elevating the proliferation and diminishing the apoptotic potential of neutrophils. In addition to the inhibition of T cell proliferation by the PDLSCs, the anti-inflammatory M2 macrophage phenotype polarization is also enhanced as the PDLSCs stimulate CD-136, interleukin-10 (IL-10) and arginase 1. 34

Similarly, GMSCs facilitate macrophage M2 polarization and also inhibit the M1 macrophages by producing prostaglandin E2 (PGE2), IL-6, and IL-10. Furthermore, they also reduce the maturation of dendritic cells that further suppresses its capability to present the antigens in a PGE2-related phenomenon, thereby dampening the inflammatory cascade. 35 The DFSCs in a similar fashion suppress bone resorption by diminishing the phagocytic activities and neutrophil extracellular trap formation and also cause M2 macrophage polarisation. 36 DFSCs also elevate the expression of anti-inflammatory cytokines such as IL-10 and suppress the concentration of the proinflammatory cytokines, thereby preventing bacterial internalisation. 37 A recent study by He et al proved that OPSCs effectively inhibited M1 polarisation. 38 The overall picture suggests that periodontal stem cells attenuate inflammation by various mechanisms as motioned above; however, the immunomodulatory capacities of these cells, which are essential participants in the modulation of immune responses to accomplish regeneration in periodontitis models, have not yet been fully explained.

## Boundless Regenerative Potential of Periodontal Stem Cells

The predictability of current treatment protocols to limit the spread of periodontal disease and facilitate regeneration is questionable. However, the principles of tissue engineering can be adapted to expedite regeneration of oral and extraoral tissues tat diversifies the application of these intimately related stem cells—the periodontal stem cells ( Table 2 ). 39

**Table 2:** Periodontal stem cells in regenerative periodontics and regenerative medicine

Stem cell	Growth factors	Carrier/scaffold used	Cell numbers achieved *In vitro in Vivo*		Model used	Regenerative outcome	Reference
PDLSCs	FGF2 RhFGF-2	Chitosan conjugated Nano HA coating	5× 10 ^4^	1× 10 ^7^	Mice-calvarial defect	• Osteogenic potential of PDLCs are enhanced • Superior hard tissue regeneration • Increased mineralization by Notch signaling	40
	TGF-β3	RGD Modified alginate microspheres	1× 10 ^6^ alginate solution	1× 10 ^6^	Mice Subcutaneous	• Enhanced tendon regeneration capacity • Higher chondrogenic and adipogenic differentiation	41
	rAd- BMP-2	Hydroxyapatite and bone grafts	2× 10 ^6^ cells/mL	2× 10 ^6^ cells/mL	Mice and canine	• BMP-2 enhances new bone formation and promotes osteogenesis	42
	BMP-2 BMP-9	1% collagen hydrogel	2× 10 ^6^	2× 10 ^6^	Canine	• Higher osteogenic differentiation	43
	IGF	Absorbable gelatine sponge Gelfoam	8× 10 ^3^ cells / cm ^2^	1× 10 ^6^	Mice	• Promotes osteogenic differentiation via osteogenesis of PDL progenitor cells	42
	1% PRP	PDLSC sheets	8× 10 ^3^ cells / cm ^2^	1× 10 ^6^	Mice	• Increases extra-cellular matrix	44
	FGF2	Amnion	3× 10 ^5^ cells	3× 10 ^5^ cells	Human	• Increased osteo, adipo differentiation	45
GMSCs	TGF-β3	Alginate microspheres	1× 10 ^6^ alginate solution	1× 10 ^6^	Mice Subcutaneous	• Enhanced regeneration capacity • Greater chondrogenic and adipogenic differentiation	46
	BMP-2	Collagen Scaffold	2mm × 3mm	2× 10 ^6^	Rats	• Higher osteogenic differentiation	47
	TNF-α	Exosomes	200 µg	1× 10 ^6^	Mice	• Higher chondrogenic and osteogenic differentiation	48
	BMP-9	Hyaluronic acid synthetic ECM	250µl	5× 10 ^6^	Dog Mini Pig	• Enhanced adipogenic and chondrogenic lineage	19
	BMP-2	Collagen membrane covering a scaffold with β-TCP	2 × 10 ^5^	8× 10 ^6^ cells / cm ^3^	Human	• Periodontal defects, enhanced osteogenic, adipogenic differentiation	49
	TGF-β	Alginate based adhesive and cross-linked hydrogel	4× 10 ^6^	4× 10 ^6^	Rat	• Higher osteogenic potential in repairing peri implantitis model	50
		Hydorgel scaffold(PuraMatrix)	1 × 10 ^6^	1 × 10 ^6^	Rat	• Maxillary alveolar defects-higher osteogenic bone formation	51
	FGF2	(PLA) 3D bioengineered scaffold Enriched with GMSCs	2× 10 ^6^ cells	2× 10 ^6^ cells	Rat	• Calvarial defects enhanced regeneration into osteocytes and adipocytes	52
IGF-1 BMP-4	Axo guard Nerve Conduits	0.5× 10 ^6^	0.5 × 10 ^6^	Rat	• Facial nerve - Enhanced neuronal and glial differentiation	53
DFSCs	BMP-2 BMP-9	HA powder	2 × 10 ^5^	2× 10 ^5^	Mice (Subcutaneous)	• Fibrous tissue formation and cementum matrix	54
	BMP-9	HA coated dental implant	5× 10 ^4^	1× 10 ^7^	Murine	• Osteogenic differentiation and periodontal ligament like tissues	55
	BMP-2	HA/ Collagen gel	2× 10 ^6^	2× 10 ^6^	Mice	• Higher differentiation into cementum like tissues – Acellular cementum	56
	IGF	Collagen nano HA/ phosphoserine biocomposite cryogel	1 × 10 ^6^	1 × 10 ^6^	Mice	• Enhanced osteogenic differentiation to bone like tissues	57
	FGF-2	Treated dentin matrix (TDM)	5× 10 ^4^	1× 10 ^7^	Canine	• Enhanced osteogenic, cementogenic, periodontal ligament tissue formation in bony defects	58
	FGF-2	TDM	5× 10 ^4^	1× 10 ^7^	Mice	• Increased formation of periodontal tissues like cementum and alveolar bone	59
	TGF- β1	Ceramic bovine bone	2× 10 ^6^	2× 10 ^6^	Mice	• Enhanced cementogenic, osteogenic and fibroblastic potential (forms cementum-PDL complex)	60
	BMP-2	Extra cellular matrix (ECM)	250µl	5 × 10 ^6^	Rat	• Enhanced bone regeneration and higher osteogenic differentiation	61
OPSCs	TGF-β BMP-2	Periosteal cell sheets	1× 10 ^6^	2× 10 ^6^	Human bone defects	• Increased bone formation by osteogenic differentiation	62
	BMP-2	OPSC cell sheets	1× 10 ^6^	2× 10 ^6^	Human	• Sinus elevation procedures- enhanced bone formation	63
	FGF-2 VEGF	Cell sheets OPSC	1× 10 ^6^	2× 10 ^6^	Mice	• Enhanced osteogenic and chondrogenic differentiation	64
	BMP-2	HA powder	2 × 10 ^5^	2× 10 ^5^	Rat	• Higher osteogenic differentiation	65
	BMP-9	HA bone graft	2× 10 ^6^	2× 10 ^6^	Mice	• Enhanced bone formation	66

Abbreviations: BMP, bone morphogenetic protein; DFSCs, dental follicle stem cells; FGF, fibroblast growth factor; GMSCs, gingival mesenchymal stem cells; HA, hydroxy apatite; IGF, insulin-like growth factor; OPSCs, oral periosteal stem cells; PDLSCs, periodontal ligament stem cells; PDL, periodontal ligament; PLA, polylactic acid; PRP, platelet-rich plasma; RGD, arginylglycylaspartic acid; RhFGF, recombinant human fibroblast growth factor; rAdBMP-2, recombinant adenovirus (rAd) encodingBMP-2; TCP, tricalcium phosphate; TGF, transforming growth factor; TNF, tumor necrosis factor; VEGF, vascular endothelial growth factor.

The potential of PDLSCs in tissue regeneration as explained by Seo et al suggested that PDLSCs could generate tissue similar to the cementum and periodontal ligament. 8 Coextensive research on PDLSC sheets exhibited the property of giving rise to structures similar to the periodontal tissues in a tricalcium phosphate matrix. The amalgamation of PDLSCs with chitosan-based scaffolds aided in bone regeneration in calvarial defect models. 67 Human DFSC sheets transplant was able to achieve optimal periodontal regeneration, including new periodontal ligament attachments, new alveolar bone development, and even a periodontal ligament–cementum complex structure. Further, DFSC sheets were more conducive to periodontal regeneration than PDLSC sheets, as observed in canine model, which might be attributable to DFSC's greater ability to adapt to the chronic inflammatory milieu of periodontitis.

Wang et al first demonstrated that transplanted GMSCs formed new bone in mandibular wounds and calvarial defects of nude mice and rats, which suggested that GMSCs can repair bone. 68 GMSCs exhibited a moderate osteogenic capability, similar to PDLSCs; however, GMSCs were stronger at forming mineralized nodules and differentiated into osteogenic, chondrogenic, and adipogenic lineages. 69 While some studies suggest that inflamed GMSCs have a reduced capability for osteogenic and adipogenic differentiation than healthy subjects, epigenetic variables linked to chronic periodontitis could impact the cell line's different orientations. The “real mesenchymal stem cells”, the OPSCs have the characteristics to differentiate into osteoblasts and chondroblasts. 25 Cacceralli et al also suggested OPSCs to be a valuable and precious alternative compared to other mesenchymal stem cells from bone marrow for tissue engineering applications in oral cavity. The extraoral applications of OPSCs need to be studied further; however, the other periodontal stem cells have widely studied in regenerative medicine for their varied differentiation capabilities.

The therapeutic potential of PDLSCs in medicine has been used for differentiation of corneal stromal keratinocytes as both PDLSCs and corneal cells are derived from the neural crest. In the personalized treatment of multiple sclerosis (MS), a comparison of PDLSCs obtained from systemically healthy patients and MS patients showed identical proliferative and differentiative potential of PDLSCs, thereby validating the use of PDLSCs in such auto-immune conditions. 70 Lee and Park demonstrated the transdifferentiation of PDLSCs into pancreatic islet cells thereby providing an alternative treatment strategy for diabetes. 71 Periodontal mesenchymal stem cells can also differentiate into cardiac muscles, skeletal muscles, endothelial and neuronal cells suggesting their therapeutic application in medical regenerative procedures.

DFSCs have a potential for neuronal differentiation as they differentiate into mature neurons and oligodendrocytes but not astrocytes. The application of DFSCs in myasthenia gravis was pioneered by Ulusoy et al, whereas the therapeutic effect of DFSCs in asthma was researched by coculturing DFSCs with the blood mononucleocytes of asthmatic patients *in vitro* . 72

GMSCs, on the other hand, have been shown to demonstrate antiageing potentials and this property can be harnessed to develop cell free treatment strategies for ageing-related and vascular disorders. 73 GMSCs have also been known to differentiate into neuronal and glial cells and this property has been harnessed in facial nerve regeneration and the management of spinal cord injuries. 74 Ansari et al reported that GMSCs encapsulated in alginate underwent osteogenic differentiation and also have chondrogenic potential without the need of additional growth factors. 75 It has been suggested that GMSCs and OPSCs exhibit similar degree of bone regeneration in defects created in rabbit models suggesting that these stem cells maybe an useful alternative in regenerative strategies. 76

## Stem Cell Derivatives in Regeneration

### E1. 3D Bioprinted Scaffolds—Self-Scaffolded Models

In scaffold-free tissue engineering, cells produce and arrange their own endogenous ECM to create a 3D structure. Scaffold-free tissue engineering, in contrast to conventional tissue engineering techniques, forgoes the use of an external scaffold material to create a 3D tissue. In 2007, a 3D bioengineered tooth—“organoid”—was made by combining dental epithelium and mesenchyme to form a complete tooth germ and it was proposed that combining in with biomaterials such as collagen can enhance the possibility of forming a bioengineered tooth in animal models. 3D spheroids can be used in a variety of culture methods to optimize the property and function of MSCs as they allow close cell–cell and cell–matrix interactions that closely resemble the microenvironment. 77 The various methods of culturing in 3D are using patterned microwells, floating culture for neurosphere formation, chitosan, ultra-low culture dishes, and poly-L-ornithine. These can be enhanced by adding growth factors like fibroblast like growth factors, lovastatins, spheroids, and mesenspheres.

PDLSCs have been successfully cultured to form 3D structures mimicking cementum and periodontal ligament using this technique. After *in vitro* cultivation, the periodontal tissue organization was evident, and it was preserved *in vivo* as well as after subcutaneous implantation in mice. These results show that PDLCs can self-assemble into an ordered cementum-PDL-like complex through scaffold-free tissue engineering. 78

Self-Assembly of a 3D spheroid culture of GMSCs can enhance the differentiation and neural stem cell properties as shown by Hsu et al, where the GMSCs were found to spontaneously aggregate into 3D spheroids with enhanced stemness and increases trilineage differentiation. 79 The GMSCs cultured in patterned microwells aggregate into 3D spheroids and have higher osteogenic potential that is augmented by addition of growth factors, while floating culture technique allows for aggregation into neurospheres and elevated neural crest markers and neuronal differentiation. The possibility of reprogramming GMSCs into neural crest like cells to differentiate into nerve cells is also possible by 3D culturing. 53

DFSCs have been seeded on 3D porous scaffolds laden with collagen-nanohydroxyapatite/phosphoserine biocompatible cryogel with osteogenic factors in the culture medium and the resultant 3D spheroids showed dynamic growth and osteogenic differentiation when implanted in mice models. 57

### E2. Use of Dental Stem Cell-Derived Exosomes in Regenerative Medicine Inverting the Disease Paradigm:

Extracellular vesicles (EVs) including ectosomes and exosomes are essential for intracellular communication as they can carry bioactive molecules such as lipids, nucleic acids, proteins, and metabolites. EVs are released as membrane bound agents from all types of cells and even found to be released from periodontal stem cells and used for treating diseases. EVs can be categorized as apoptotic vesicles, microvesicles, and exosomes. EVs are successful in treating a variety of disorders by encapsulating and conveying essential bioactive components (e.g., proteins and nucleic acids) to affect the phenotype of target cells.

PDLSCs produce exosomes that harbor the potential of repair and regeneration, which can induce angiogenesis, alleviate neurological diseases, and reduce the inflammatory microenvironment. 80 In periodontitis, they can be used to induce osteogenesis and enhance bone regeneration as seen by Pizzicannella et al, in lesions of the rat skull, where addition of PDLSC-derived exosomes to a 3D collagen membrane and polyethyleneimine scaffold, showed bone regeneration. 81 Further, it has been observed that human PDLSC-derived exosomes promote osteogenesis by the expression of their exosomal miRNAs *in vitro* . 82 Similarly, GMSC-EVs in periodontitis models in rats show periodontal regeneration by delivery of mIR-120b to inhibit osteoclastogenic activity of PDL cells by targeting the Wnt5a-mediated RANKL pathway. 48

DFSCs-sEVs were found to greatly increase PDLSC migration, proliferation, and osteogenic differentiation and regeneration of periodontal tissue by stimulation of the p38-MAPK signaling pathway. Small EVs from DFSCs provide biochemical cues for periodontal tissue regeneration. 83 There is potential to use OPSC-derived EVs in future for bone regeneration as they are more committed to an osteogenic lineage. However, to achieve an optimal periodontal regeneration of the intricate structures in the periodontium, more research is still required to identify ideal and standardized sources of EVs, their effective concentration, frequency of treatment, and suitable scaffolds or delivery routes. Better insight into the therapeutic potential of periodontal stem cells derived-EVs would provide more reasonable options for the future treatment of periodontal diseases.

## Clinical Application of Stem Cells in Human Periodontal Regeneration

The use of stem cells cultured in scaffolds has shown promising results *in vitro* and animal model studies. The subsequent application of these stem cells needs to be explored whether it can bring about regeneration as previous clinical trials have been performed in this regard to achieve periodontal regeneration using autologous transplanted stem cells from the periodontal complex. PDLSC sheet transplants in humans have shown no adverse effects and have generated a reduction in probing depth; however, this was not significant. Further, a clinical trial with autologous PDLSC transplants in periodontitis patients had shown improvement in periodontal parameters as seen by reduction in pocket depths and bone regeneration. Transplants of PDLSC sheets mixed with granules of β-tricalcium phosphate in bone defects have also shown no adverse effects till 6 months. 84 PDLSCs implanted in animal models show superior periodontal regeneration in the form of greater cementum, bone, and PDL formation. A recently concluded clinical trial on animal model showed that when the PDLSC was combined with collagen membrane on fenestration defects, it showed a greater cementum formation but no difference in bone formation when applied without the membrane. 85 Similarly, in furcation defects when PDLSCs were combined with hyaluronic acid sheets, it resulted in greater cementum, bone, and PDL formation than controls. 86 Further, PDLSC sheets combined with β-tricalcium phosphate in infrabony defects resulted in nearly complete regeneration of periodontal tissues. 87 Over all, it can be deciphered that the application of PDLSCs in animal models is predictable for cementum formation; however, the results are conflicting when it comes to bone regeneration.

Use of GMSC in future clinical trial seems to be a promising approach as systemically administered GMSCs have the ability to home to the injury site and differentiate into osteoblasts, cementoblasts, and periodontal ligament fibroblasts as tested in animal models. In class III furcation defect animal models, GMSC sheets significantly have enhanced the regeneration of periodontal tissues. 51 Combination of the GMSC with HA gel has also showed a significant regeneration in porcine model by exhibiting formation of newly formed bone and PDL fibres. 88 GMSC human clinical trials have also been employed in treating periodontal defects while embedding these cells in collagen scaffolds mixed with β-tricalcium phosphate, thereby reducing probing depth, attachment gains, and alveolar bone gain as seen in 6 months follow-up. 49

DFSC sheets implanted in animal models have shown to regenerate whole periodontal tissue as observed by formation of complex–periodontal ligament like structure within a month. Implanting into periodontal irregularities *in vivo* , DFSCs show a better capacity for cementum and periodontal attachment healing than PDLSCs due to higher involvement of extracellular matrix. 29 89 The DFSCs work by providing an ideal microenvironment for the growth of PDLSCs and act as a scaffold.

Human OPSCs, on the other hand, have shown promising results *in vivo* when transplanted in human intrabony periodontal defects and in sinus elevation procedures for forming bone. 63 There are enhanced osteogenic properties of OPSCs when transplanted with growth factors and enriched with collagen scaffolds. 62

## Conclusion

Multiple stem cell populations including PDLSCs, GMSCs, DFSCs, and OPSCs coexist in close proximity and still stay in function in spite of continual remodeling and inflammation in the periodontal complex. Periodontal stem cells have a strong interaction with the inflammatory milieu, as well as the ability to modulate the immune system, making them lucrative candidates for cell treatment in periodontitis and inflammatory disorders. The periodontal stem cells are one-of-a-kind, with a variety of morphologies and multipotency, both *in vitro* and *in vivo* . Each stem cell population's differentiation capacity is diverse, and it can repair bone, neurons, and tendons in addition to mesenchymal tissues in the oral cavity. The PDLSC are thought to be harboring diverse regenerative potential within the oral cavity; however, they are more differentiated cells. The DFSCs in contrast exhibit greater propensity for extraoral tissue differentiation as shown by higher expression of embryonic markers like Oct4 and Nanog. GMSCs are touted to be the stem cells with greater accessibility and higher differentiation capacity as seen by various clinical models and 3D bioprinting studies. The use of GMSCs for nerve regeneration is promising in future. The OPSCs, on the other hand, need to be studied further to understand their behavior both *in vitro* and in clinical human models. Although it would not be appropriate to state the superiority of one stem cell type over another, it is plausible that therapeutic application of these stem cells to regenerate hard and soft tissues and alleviate degenerative diseases may become a reality in the future. To this regard, additional prospective and long-term trials are needed to determine the true characteristics of each population and how they might be used for exogenous MSC grafting, 3D bioprinting, specific exosomal derived vesicles, and cell homing in periodontal tissue engineering.
